# Supplementary material for: Are carbon emissions trading and green financial instruments synergistic? -Comprehensive quantitative research based on content analysis
Source: PLoS One. 2024 Mar 7;19(3):e0298601. doi: 10.1371/journal.pone.0298601 (PMC10919601; doi:10.1371/journal.pone.0298601)
Supplement: S1 Data — (ZIP) [file pone.0298601.s001.zip › Data/地级市Z_F工作报告.zip!/║╙─╧/╞╜╢Ñ╔╜╩╨/└┤╫╘╣1⁄2╓┌║┼í╛┬φ┐╦╩2╛▌═°í┐_2008.docx]

各位代表：

现在，我代表市人民政府，向大会作政府工作报告，请予审议，并请各位政协委员提出意见。

一、2007年工作回顾

2007年，市人民政府在省委、省政府和市委的正确领导下，认真贯彻落实科学发展观，积极构建和谐社会，团结带领全市人民开拓进取、扎实工作，圆满完成了市八届人大五次会议确定的各项任务。

——总体实力再上台阶。初步核算，全市生产总值821.5亿元，比上年增长15.4%，总量由全省第7位上升到第6位。其中，一、二、三产业分别实现增加值88.5亿元、514.7亿元和218.3亿元，增长5.7%、18.1%和13.4%。人均生产总值达到16983元，增长15.4%。地方财政一般预算收入50.1亿元、支出84.8亿元， 分别增长24%和24.6%，收入规模继续保持全省第3位。

——三大需求明显增长。全社会固定资产投资365.3亿元，增长34%，其中城镇投资311.4亿元，增长32.1%;年末金融机构各项贷款余额406.3亿元，增长22%。社会消费品零售总额199.1亿元，增长18.1%。外贸出口3.17亿美元，增长16%。

——人民生活继续改善。城镇新增就业10万人。城镇居民人均可支配收入11715元，农民人均纯收入3733元，分别增长18.4%和18.5%。年末城乡居民储蓄存款余额425.1亿元，增长11.8%。城市居民住房成套率87.1%，提高1个百分点。

——社会事业全面进步。科技进步对经济增长的贡献率达到47%。教育“两基”工作顺利通过国家验收。在全省率先建立了高中阶段贫困生救助制度和全民医保制度。市博物馆、市文化艺术中心主体基本完工。成功举办市七运会暨第十一届全民健身节。人口自然增长率控制在5.5‰以内。

2007年，市政府主要做了以下工作:

(一)突出项目带动，大力推进经济转型。坚持以项目建设促进产业升级，加快发展方式转变。工业结构进一步优化。全年城镇以上单位完成工业投资161.6亿元，增长19.6%。舞钢新建100万吨宽厚板生产线、神马年产3万吨尼龙66切片连续聚合技改、平高东芝二期扩建、姚电四期2×60万千瓦机组扩建等23个重点工业项目建成投产，年产240万吨的平煤七星选煤厂、年产240万吨的平煤十一矿选煤厂、香山(平西)500千伏超高压输变电工程等8个项目开工建设。全市规模以上工业实现增加值338.8亿元，增长23.5%，其中非煤产业占64.6%，提高0.5个百分点;高技术产业占2.5%，提高0.2个百分点。30户重点工业企业主营业务收入达到699.7亿元，增长36.9%，其中舞钢公司突破110亿元，平煤集团突破340亿元。自主创新能力明显增强。全年科技投入1.15亿元，增长60.5%。由平煤田庄选煤厂完成的“新型双流态微泡浮选机研究开发和应用”项目获国家技术发明二等奖。15项成果获省科技进步奖，其中一等奖2项。新建省级企业技术中心2家、省级企业工程技术研究中心5家，新认定省级高新技术企业9家、高新技术产品11个。神马集团和平高电气被确定为河南省首批创新型试点企业，平高集团入选第二批国家级创新型试点企业。天瑞集团在全国首家拥有火车轮铸造自主知识产权和专利技术。新增中国名牌产品2个、省级名牌产品8个，平高电气“PG”商标被认定为中国驰名商标。新兴服务业不断壮大。文化旅游产业进一步发展。郑石高速公路全线通车，石人山生态旅游开发步伐加快。全年接待国内外游客439.8万人次，增长23.1%;旅游业总收入18.8亿元，增长24.8%;文化旅游产业增加值占第三产业比重由14.8%提高到15.3%。房地产业发展加快。全年房地产开发投资24.8亿元，增长65.7%。现代物流、金融保险等其他服务业也都有新的发展。

(二)围绕农民增收，切实加强“三农”工作。继续巩固农业基础地位，全面落实支农惠农政策，新农村建设扎实推进。全年财政支农支出6.3亿元，增长42.6%。现代农业建设取得新成效。全年粮食总产193.9万吨，增长6.3%，再创历史新高。新增无公害农产品39个，小麦优良品种覆盖率达 到99%。新增标准化养殖小区86个、规模化养殖场318个。新增市级农业产业化龙头企业21家、省级重点龙头企业9家、农民专业合作经济组织53家。完成农民职业技能培训14.6万人、科技培训41.2万人，新转移农村富余劳动力14.7万人，外出务工总量达到100万人，当年实现劳务经济总收入100亿元。农村生产生活条件又有新改善。全市新农村建设五年规划顺利实施，102个市级示范村新村建设规划基本编制完成。全年新增有效灌溉面积3.2万亩，治理水土流失面积78.3平方公里，新增农机总动力17.5万千瓦。新解决11.2万农村人口的饮水安全问题，新建改建农村公路1115公里，新建户用沼气池4.1万座，解决农村无电户1.4万户，实现了户户通电。解决和巩固温饱贫困人口4.1万人。县域经济发展实现新突破。6县(市)完成生产总值549.6亿元，增长17.1%，占全市的比重由65.2%提高到66.9%。10个县(市、区)财政收入全部超2亿元，最高的突破8亿元。舞钢市和鲁山县分别被省政府评为县域经济综合实力强县和先进县。叶县被命名为“中国岩盐之都”。

(三)加大“双创”力度，全面提升城镇化水平。深入开展国家园林城市、卫生城市创建活动，城市品位和档次不断提升。全市城镇化率达到38.8%，提高1.8个百分点。中心城区功能日趋完善。新城区完成各类投资20.6亿元，新建、续建道路18条，框架拉大到25平方公里。会议中心建成投用，湖光小学开始招生，第七中学基本建成，第二安置小区主体完工。老城区高阳路三期、开源路南延、凌云路、东风路建成通车，新华路北延立交桥主体工程完工，工人文化宫升级改造、联盟路连片开发及和平路西段商业步行街改造进展顺利，湛河治理四期工程开工建设，采煤塌陷区综合整治加快推进。大力实施园林绿化工程，新建绿地208万平方米，城市建成区绿地率和绿化覆盖率分别达到28.3%和34%。县城和小城镇建设得到加强。出台了小城镇基础设施建设指导意见，督促各县(市)重点镇完成了18条道路和一批游园绿地、环卫设施建设。舞钢市成功创建为国家园林城市。城市管理更加精细。根据国家卫生城市标准，制定并实施了公共场所行业卫生、集贸市场等8个方面的执行细则。在市区主干道和重点区域增加保洁人员、增派执法人员、增设垃圾箱，延长保洁时间，对乱停乱放、占道经营进行了综合整治，城市面貌有新的改观。

(四)深化改革开放，不断增强发展活力。推动体制机制创新，全面扩大对外开放。各项改革深入推进。继续精简行政审批事项，压缩办理时限，全市45.6%的审批项目办理时效位居全省前列，比第二名高出27.4个百分点。完成国有中小企业改制28家，全市302家国有企业改制面累计达93%， 其中国有工业企业改制面99.4%。企业学校移交工作基本完成。农村信用社改革取得实质性进展，成功兑付央行票据7.67亿元。城市信用社改革继续深化。邮政储蓄银行平顶山分行正式挂牌。工资收入分配制度改革有序展开。水管体制改革全面启动。农村公路养护体制改革加快推进。其他领域的改革也取得了新进展。对外开放步伐加快。通过参加豫港投资贸易洽谈会、中博会、华创会，举办建市五十周年经贸洽谈会和宁波招商会，大力开展招商引资工作。全年新批外商投资企业10家;实际利用外资9090万美元，增长56.5%，创历史最好水平;实际到位市外资金140.2亿元，增长23.4%。引导企业开展国际合作，平煤集团与津巴布韦国家电力公司签署了联合开矿协议，平高集团与叙利亚输配电公司签订了输变电线路成套项目总承包合同，天瑞集团与美国KKR公司、IFC国际金融公司合作建设水泥生产项目。非公有制经济快速发展。落实扶持政策，优化发展环境，加强银企合作，非公有制经济实现增加值413亿元，增长15.8%，占生产总值比重达到50.3%;完成税收36.8亿元，增长24.6%。

(五)狠抓节能减排，加快建设资源节约型和环境友好型社会。实行严格的目标责任制，综合运用多种手段，促进节能减排任务的落实。淘汰落后产能力度加大。关闭了8家小煤粉厂、珍珠岩厂和所有水泥立窑生产线，关停小火电机组46.2万千瓦。拆除黏土砖瓦窑厂474个，平整复垦土地9765亩。发展循环经济成效初显。引导企业不断增加投入，实施了煤层气发电、矿井水综合利用、煤矸石烧结砖、粉煤灰制品等一批循环经济项目。全市新型墙材产能达到32亿标块，工业固体废弃物综合利用率提高到70%。环境综合整治和生态建设得到加强。对重点行业、重点企业的主要设备、污水处理系统、除尘系统进行了节能减排技术改造。深化了矸石山综合治理、秸秆禁烧工作。6县(市)污水处理厂、垃圾处理场建成投用。全面启动林业生态市建设，完成大面积造林18.2万亩，新建和完善农田林网130万亩，通道绿化1101公里。鲁山县、石龙区完成省级林业生态县创建任务。全市万元生产总值能耗下降4.5%;化学需氧量、二氧化硫排放量分别下降12.3%和4.8%;城区大气环境质量优良天数达到313天，比上年增加52天;出境水质达标率84.7%;城市集中饮用水源地水质达标率100%。

(六)着力改善民生，积极构建和谐社会。认真解决关系人民群众切身利益的问题，“十大实事”全部落实。就业和社会保障工作扎实推进。完善扶持政策，加强就业援助，积极扩大就业。全年发放小额担保贷款6012万元，建成创业孵化园区3个，帮助下岗失业人员再就业3.9万人，其中困 难群体就业1.4万人。在上年基本消除“零就业家庭”的基础上，又消除新增的“零就业家庭”167户。社会保险覆盖面进一步扩大，全市企业养老、医疗、工伤、女工生育保险参保人数分别增加2.3万、2.5万、0.6万和2.3万人。城乡低保得到落实，为8.6万城市低保对象和12.4万农村低保对象发放低保金1.3亿元。爱心救助超市救助城乡困难群众5.6万多人次。新建和改扩建乡(镇)敬老院73所，农村五保对象集中供养率达到30%。全民医保制度初步建立，新型农村合作医疗参合农民318.6万人，参合率87.2%;城镇居民合作医疗参合人数41.1万人，参合率85.7%。低收入家庭住房保障得到加强，棚户区改造开工143万平方米，竣工65万平方米;经济适用房建设开工15.6万平方米，竣工2.8万平方米;发放廉租房补贴145万元，基本解决了1056户困难家庭的住房问题;发放住房公积金贷款3.5亿元，为3300户住房困难家庭提供了资金支持。社会事业全面发展。落实农村义务教育“两免一补”资金1.57亿元，资助家庭困难学生47万人次。消除农村中小学危房9.6万平方米。更新配置课桌凳16.9万套。加快发展医疗卫生事业，完成了24所乡(镇)卫生院改造。实施广播电视“村村通”和农村电影放映、文化信息资源共享、文化下乡等工程，成功举办首届社区文化艺术节、特色文化活动周等活动。郏县大铜器、宝丰酒酿造技艺入选第二批国家级非物质文化遗产保护名录。体育事业不断发展，在第六届全国城市运动会上我市选手获两枚金牌，实现历史性突破。加强社区建设，中心城区87个社区硬件建设全部达标。认真做好人口和计划生育工作，稳定了低生育水平。精神文明和民主法制建设继续加强。深入开展社会主义荣辱观教育和精神文明创建活动，新命名省级文明单位40家。舞钢市成为全省首批命名的6个文明城市之一。不断加强法治诚信服务型政府建设，依法行政水平明显提高。自觉接受人大及其常委会的法律监督、工作监督和人民政协的民主监督，认真办理人大代表建议和政协提案。民兵和预备役建设及优抚安置工作扎实开展，国防教育、双拥共建、人民防空工作深入推进，连续第三次荣获全国“双拥模范城”称号。社会信用体系建设得到加强。廉政建设和反腐败斗争取得新成效。安全生产和社会大局保持稳定。认真执行安全生产的各项法律法规，严格落实企业的安全主体责任和政府的监管主体责任，全市安全生产形势总体平稳。深入开展产品质量和食品安全专项整治，全年没有发生重大食品药品安全事件。加强信访、市长热线工作，群众诉求渠道进一步畅通。加大社会治安综合治理力度，推进治安防控体系建设，依法打击各类犯罪活动，维护了社会稳定。

各位代表，过去的一年，是我市现代化建设取得显著成绩的一年。在宏观调控趋紧、区域竞争加剧、产业升级加快的大环境下，我们牢牢把握项目建设、招商引资、优化环境等关键环节，加快结构调整和产业升级，经济发展既保持了较好的质量和效益，又保持了较高的增长速度，发展方式更加科学;加快新农村建设和农业结构调整，既注重提高农业综合生产能力，又注重解决与农民切身利益密切相关的问题，农业基础更加巩固;加快新城区开发和老城区改造，既注重基础设施建设，又注重提高管理水平，城市功能更加完善;在加快经济建设的同时，更加重视教育、文化、卫生等社会事业的发展，解决了一大批关系人民群众切身利益的热点、难点问题，经济社会发展更加和谐。

各位代表，这些成绩的取得，是省委、省政府和市委正确领导的结果，是人大、政协积极支持的结果，是全市广大干部群众共同奋斗的结果。在此，我代表市人民政府，向工作在全市各条战线上的工人、农民、知识分子、干部、人民解放军驻平部队指战员、武警官兵和公安民警致以崇高敬意！向关心、支持 我市现代化建设的各界人士表示衷心感谢！

在肯定成绩的同时，我们也清醒地看到，我市经济社会发展中还存在一些突出矛盾和问题。主要是:产业结构不尽合理，资源开采和能源消耗型工业比重大，第三产业发展滞后;中小企业自主创新能力较弱，高新技术产业占工业的比重较小;土地、资金约束加剧，环境保护和节能降耗压力加大，发展方式亟待进一步转变;城市基础设施不尽完善，规划和管理仍需加强;低收入群体在住房、医疗、教育等方面还有不少困难，构建和谐社会的任务依然艰巨;政府职能转变还不到位，一些政府工作人员服务意识不强、工作效率不高，发展环境仍需改善。此外，部分经济指标增速下滑、安全生产形势严峻、农业增产和农民增收难度加大等问题也较突出。我们必须采取更加有力的措施，切实解决这些问题，决不辜负全市人民的期望！

二、2008年工作总体要求和目标任务

党的十七大胜利召开，必将推动全国经济社会进入新一轮发展高潮。与全国、全省一样，我市面临的是一个机遇与挑战并存、机遇大于挑战、总体上有利于加快发展的环境。经济全球化深入发展，国际投资和贸易持续活跃，国际国内产业转移步伐加快，为我市加快发展创造了良好的外部环境;国家加大对“三农”、结构调整、自主创新、节能减排和改善民生的投入，全面落实促进中部崛起的具体政策，出台促进资源型城市可持续发展的意见，以及我省逐步实施促进中原城市群发展的具体政策等，为我市加快发展提供了难得的政策机遇;全市上下干事创业的氛围日益浓厚，一批新上的重大结构调整项目逐步形成生产能力，为进一步加快发展奠定了好的基础。我们要以奋发有为的精神状态和求真务实的工作作风，创造性地开展工作，努力促进经济社会又好又快发展。

今年政府工作的总体要求是:以党的十七大精神为指导，深入贯彻落实科学发展观，围绕“走在全省前列、率先实现崛起”的战略目标，按照好字优先、好中求快的要求，进一步解放思想，继续深化改革扩大开放，加快推进自主创新，着力优化经济结构和提高发展质量，切实加强节能减排和生态环境保护，更加重 视改善民生和促进社会和谐，努力推动经济建设、政治建设、文化建设和社会建设取得新成效，奋力开创各项工作新局面。

今年政府工作的主要预期目标是:生产总值增长12%以上;地方财政一般预算收入增长15%以上;全社会固定资产投资增长18%以上;社会消费品零售总额增长15%以上;进出口总额增长15%以上，实际利用外资增长25%以上;居民消费价格涨幅控制在4.8%左右;城镇居民人均可支配收入增长10%以上，农民人均纯收入增长8%以上;城镇新增就业7万人以上，城镇登记失业率控制在4.5%以内;人口自然增长率控制在6.5‰以下;单位生产总值能耗降低5%以上;化学需氧量、二氧化硫排放量分别下降2%和2.7%以上。

在完成上述目标的同时，我们将积极筹措资金，着力办好事关群众切身利益的“十大实事”:一是加大对农民的补贴力度;二是切实加强农村基础设施建设;三是大力发展文化体育事业;四是进一步提高义务教育保障水平;五是继续改善群众医疗卫生条件;六是扎实推进城市低收入家庭住房保障工作;七是认真做好就业再就业和农民工培训工作;八是加强社会保障和对城乡困难群众的救助;九是努力保障食品药品安全;十是积极推进污染防治。使全市人民更多地享受改革发展的成果！

三、努力促进经济发展方式转变

大力推进经济转型，切实提高经济整体素质和市场竞争力，加快建设新型能源和工业基地。

(一)继续加快基础产业和基础设施建设。进一步优化投资结构，提高投资效益，着力抓好一批带动能力强的重大项目建设，夯实发展基础。加快能源原材料生产基地建设。强力推进大中型矿井建设和煤矿安全技术改造，完成平煤十一矿改扩建、梨园矿宁庄井和香山公司矿井升级改造等项目，加快平煤朝川矿升级改造续建项目建设，做好平煤夏店矿项目前期工作，争取新增煤炭生产能力270万吨。努力扩大选煤、洗煤生产能力，完成平煤七星选煤厂技改和十一矿洗煤厂项目建设。全面完成地方煤矿技改矿井技术改造任务。加快建设大容量火电机组和煤矸石综合利用发电项目，开工建设平顶山第二发电厂一期2×100万千瓦、郏县耀阳煤矸石综合利用1×30万千瓦发电项目。加大电网建设力度，完成香山(平西)500千伏超高压和汝州北、九里山、叶县蓝光3个220千伏输变电工程及1000千伏特高压交流示范工程(平顶山段)项目建设。推动钢铁产业技术改造，加快舞钢新宽厚板生产线调整和热处理项目建设，做好中加三期工程前期工作。大力发展建材产业，重点支持水泥骨干企业加快发展，形成质量效益位居全省前列的新型干法水泥生产基地;完成宝丰洁石建材年产1400万平方米陶瓷砖项目。加快交通运输体系建设。确保太澳高速(平顶山段)、范辛铁路(平顶山段)年内建成通车，开工建设叶舞高速、新城区至宝丰快速通道和焦柳线洛阳至襄樊电气化(平顶山段)等项目，积极推进汝登高速(平顶山段)、孟宝铁路复线等项目前期工作。加快重点水利项目建设。完成燕山水库建设和龙兴寺水库除险加固项目，加快建设孤石滩灌区节水续建配套工程，做好南水北调(平顶山段)项目前期工作。继续实行项目法人负责制度、市级领导联系重点项目制度和工作督查制度，积极为项目建设创造良好环境。

(二)大力推进产业结构优化升级。从创新体制机制、完善和落实扶持政策入手，加大工作力度，提升产业竞争力。加快企业联合重组和上市步伐。积极引进战略投资者，推进企业联合重组。重点支持平煤、神马联合发展煤盐化工，建设化工产业聚集区;支持神马与跨国公司战略合作，带动化纤行业升级改造 ;支持舞钢与中加合作，打造牢固的钢铁产业链;支持平高与国内大企业联合重组，扩大高端市场份额;支持天瑞与其他水泥企业联合重组，增强区域市场控制能力;支持宝丰酒业改制重组、扩能升档，重塑中国名酒品牌形象。加大企业上市的培训辅导力度，加快天瑞集团、中加公司上市步伐，争取巨龙公司、梦想公司、海明公司等企业早日上市。继续落实促进重点工业企业加快发展的意见，做大做强重点企业，力争30户重点工业企业主营业务收入达到800亿元以上。培育新兴接续产业和高新技术产业。着力推进煤盐化工项目建设，完成神马20万吨尼龙66盐成套装备、双20万吨氯碱及神马汇源双10万吨氯碱等项目，争取开工平煤年产50万吨甲醇、30万吨PVC及配套25万吨烧碱等项目，加快平煤年产100万吨联碱等项目建设，积极推进神马年产60万吨离子膜烧碱及60万吨聚氯乙烯树脂易地搬迁等项目前期工作。支持装备制造业发展，完成平高超高压组合电器重大装备本地化、超高压开关设备智能化升级及配套、平煤机高端液压支架及乳化泵站技改等项目，开工建设新平高特高压超高压开关重大装备基地、天瑞铁路特种机车高速重载合金钢配件配套扩能、平煤机液压支架计算机电液控制系统产业化等项目。大力发展高新技术产业，申报省级高新技术企业3家、高新技术产品5个，高技术产业增加值增长15%以上。引导产业集聚发展。以现有的开发区、工业聚集区为主体，加快基础设施和服务体系建设，引导工业企业向产业聚集区集中、新上化工项目向化工产业聚集区集中、新上装备制造业项目向开发区集中。设置项目进入门槛，既要以大代小，更要以先进代落后;既要产业的集中度，更要产品的关联度;既要节能减排达标，更要追求单位面积投资强度和同行业投入产出比最大化。大力发展以旅游业为龙头的现代服务业。认真落实加快旅游业发展的政策措施，加强规划，整合资源，拉长旅游产业链，带动服务业全面发展。抓住郑石高速通车的有利时机，大力推进石人山生态旅游区开发建设，把石人山景区、佛泉寺景区和温泉群打造成伏牛山核心景区的龙头、著名的佛教文化圣地、国内一流的温泉度假区。加快香山景区保护性开发建设步伐。积极推进中国“盐湖”度假村建设前期工作。加大旅游宣传推介力度，组织好“魅力平顶山、旅游新天地”全国摄影大赛。大力发展现代物流、金融保险、交通运输、信息服务、商务中介等生产性服务业，提升水平，扩大规模。积极扩大城市消费，大力开拓农村消费市场。实施“家电下乡”工程，对农民购买规定类家电，按销售价格给予13%的直接补贴。培育大型房地产企业，推动建筑企业加快发展。

（三）扎实推进社会主义新农村建设。坚持把“三农”工作作为重中之重，以发展现代农业为重点，促进粮食稳定增产、农民持续增收。强化和完善农业支持政策。筹措资金2.55亿元，继续实施对种粮农民直接补贴、优质专用小麦良种补贴、农村劳动力转移培训补贴、测土配方施肥补贴，增加农业生产资料 综合直补和农机具购置补贴，落实奶牛和生猪扶持政策。继续实行小麦托市收购政策。大力发展现代农业。加强耕地特别是基本农田保护，治理水土流失面积70平方公里，新增有效灌溉面积3万亩，确保粮食产量不低于160万吨。切实抓好“菜篮子”工程。推广叶县畜牧业标准化养殖经验，加快无公害生猪生产基地、优质奶源基地、优质肉牛基地建设。新建生猪标准化养殖小区100个、奶牛标准化养殖小区20个，新增奶牛2.5万头，力争用两年时间使全市奶牛发展到10万头以上。加大对农业产业化龙头企业的扶持力度，引进1~2家较大规模的畜产品加工企业，新增市级龙头企业20家，再有3家企业达到省级重点龙头企业标准。新增农民专业合作经济组织50家。加快农业科技推广体系建设，积极推进农业科研成果转化。加强农产品质量安全工作，建成市农产品质量检测中心，健全县级农产品质量安全检验检测站。逐步推行农产品产地准出和市场准入制度。抓好市、县两级农业科技信息服务中心和25个乡(镇)信息服务站建设。完善市、县、乡三级动物疫病防控体系，做好重大动物疫病防控和应急工作。分类推进社会主义新农村建设。全面推进新农村经济发展规划和新村建设规划的编制及已编规划的实施工作。开展以中心村和重点小城镇为主体的村(镇)体系规划与建设，积极探索整乡推进的路子。同时，对全市所有行政村分类排队，制定分类推进的具体实施方案。对困难村，制定市直单位对口帮建计划，实行副县级以上干部带队驻村帮建。增加并整合支农资金，加快农村道路、饮水、沼气、广播电视等基础设施建设，用三年时间有效改善环境状况和村容村貌，推动社会主义新农村建设取得突破性进展。筹措资金3.19亿元，新改建农村公路650公里，再解决10万以上农村人口的饮水安全问题，新建户用沼气池3万座，再建成500个农村便民超市，实现595个20户以上自然村通广播电视，启动“井井电灌、亩亩增收”建设工程。加强老区建设和扶贫开发工作，完成60个贫困村的整村推进和400户深山区散居户、独居户的搬迁任务，确保全市5.5万农村贫困人口脱贫。扶持104个行政村实施农民健身工程。筹措资金2885万元，完成农民工务工技能培训5.5万人，新转移农村劳动力6万人。落实移民扶持政策，做好移民安置工作。着力提升县域经济发展水平。出台促进县域经济发展的意见，创新激励机制，完善考评体系，加强分类指导，引导企业向产业聚集区集中、生产要素向优势企业集中、人口向城镇集中，推动县域经济结构优化、发展转型。继续推进舞钢市城乡一体化试点工作。力争县域经济占全市生产总值比重和对财政收入的贡献率均提高1个百分点以上。

(四)着力提高科技创新能力。坚持把提高科技创新能力作为推进结构调整的中心环节，加快创新体系建设。加大科技项目研究开发力度。认真落实中长期科技发展规划纲要，完善促进科技创新的机制和政策。实施好重大科技专项，着力突破一批制约我市重点产业、重点领域发展的关键技术。组织实施16项成 熟技术的转化推广、60项实用技术的成果鉴定，完成省级以上成果鉴定30项。提高企业创新能力。加强研发中心建设，推进产学研结合，新建省级企业技术中心3家，申报省级企业工程技术研究中心7家、省级重点实验室1个。大力实施名牌战略，新增中国名牌产品2个、省级名牌产品5个。出台标准化战略实施意见，鼓励和支持我市的优势企业制定或参与制定行业标准、国家标准甚至国际标准，引导更多的企业采用先进标准生产。加强知识产权保护。落实知识产权政策法规，实施知识产权战略，全年申请专利300件以上。大力发展知识产权经济，扶持具有自主知识产权的技术、标准和品牌，促进自主知识产权成果产业化。

(五)切实加强城镇建设和管理。认真贯彻城乡规划法，进一步加强城乡规划管理。开展平宝、平叶一体化规划工作。按照国家园林城市和卫生城市标准，大力推进城镇基础设施建设、园林绿化工程和环境卫生整治，确保“双创”指标如期完成。力争城镇化率提高2个百分点。加快老城区建设和改造步伐。完成开源路铁路立交桥改造及连线和新华路北延工程。继续抓好湛河治理四期工程，加快推进西杨村河和城东河治理。实施高阳路一、二期配套和矿工路改造工程。分期对市区湛河四桥进行改造。开工建设市垃圾无害化处理场。基本完成采煤塌陷区综合整治。启动15个“城中村”的开发建设，积极吸引有实力的房地产开发企业参与规模改造。研究制定优先发展城市公共交通的实施意见，加强城市公共交通基础设施建设。完善新城区服务功能。研究出台完善配套设施、提升新城区吸纳能力的意见，加快推进市政道路、供水、供电、燃气、通讯、城市公交、环卫设施、园林绿化等基础设施建设，完善学校、医院、商业、金融、文化等配套服务功能，提高综合承载能力。加大县城和小城镇建设力度。认真搞好县城规划、建设和管理，吸引开发商参与建设和改造。积极开展园林县城、卫生县城等各类创建活动。突出抓好31个重点镇建设，各县(市)再有1~2个小城镇建设通过达标验收。提高城市精细化管理水平。完善城市管理体制，落实各级城管责任制，加强部门协同，开展综合执法。突出抓好背街小巷、城郊村庄、铁路沿线等部位的综合治理，坚决制止乱搭乱建行为。深入开展市容环境、市政设施、园林绿化、公共交通等方面的精细化管理，提高城市管理水平。

(六)继续深化改革扩大开放。加大改革开放力度，为科学发展提供体制机制保障和强大动力。积极推进各领域的改革。全面完成国有中小工业企业改制。基本完成已进入改制程序的13户其他各类国有企业改制。进一步深化农村信用社改革。完成城市信用社组建商业银行工作。支持农业银行股份制改革。全面 推进集体林权制度改革，集体林和宜林地明晰产权、确权发证率达到85%%以上。积极探索农村土地流转的有效途径，稳妥推进土地适度规模经营。加快发展开放型经济。积极组团参加第三届“中博会”、第五届“河南国际投洽会”、“厦洽会”、“深圳高交会”等重大招商会，筹办华侨华人中原经济合作论坛、首届中国盐商论坛暨投资洽谈会，适当时机在沿海发达地区组织一次大型招商活动。更加注重招商项目的针对性和先进性，实现从招商引资向招商选资转变。加强对签约项目的跟踪服务，提高外资实际到位率。优化出口产品结构，提高出口产品档次和附加值。鼓励有条件的企业“走出去”，开展资源开发、境外加工、生产制造等经营活动。加快发展对外承包工程与劳务合作，不断扩大外派劳务规模。进一步壮大非公有制经济。出台促进非公有制经济发展的意见，在放宽市场准入、改进金融服务、促进技术创新、健全服务体系、加强人才培养等方面，加大扶持力度。鼓励非公有制企业参与国有资本调整和战略重组。加强中小企业信用担保体系建设，抓紧组建创业投资担保公司，为企业搭建融资平台。重点扶持100户非公有制企业和中型企业。力争非公有制经济占全市生产总值比重和对财政收入的贡献率均提高2个百分点以上。

四、切实加强文化建设

抓住国家、省推动文化大发展大繁荣的机遇，充分挖掘文化资源，搞好整体策划，打造文化名片，提升文化软实力。

(一)大力发展文化事业。加大公益性文化基础设施建设投入，市博物馆、文化艺术中心基本竣工。加强县级文化馆、图书馆、乡(镇)文化站和农村文化大院建设，新建乡(镇)综合文化站6个、农家书屋100个。完成3个县级文化信息资源共享工程分中心建设和510个行政村文化信息资源共享工程的升级改造，各县(市、区)至少建成一个城镇数字电影广场，全市60%的行政村一月放映一场公益性电影。落实省“百部流动舞台千场演出送农民”活动。精心打造舞台艺术精品，繁荣发展新闻出版、广播影视、文学艺术和哲学社会科学。办好第六届中国曲艺节、中国·宝丰第四届魔术文化节和市首届农民艺术节。加强文物和非物质文化遗产保护、开发与利用，加快清凉寺汝官窑大遗址保护工程进度。启动中国观音文化之乡申报工作。积极开展全民健身活动，提高业余训练水平。

(二)加快发展文化产业。重点发展以宝丰魔术为代表的演艺业、以汝瓷为代表的陶瓷业、以文物景点为代表的文化旅游业等优势产业，培育一批龙头文化企业，继续打造好“宝丰文化现象”这一品牌。加快宝丰赵庄魔术大观园、马街民俗风情园、舞钢冶铁文化园等特色文化产业园区的建设和发展。扶持网络服务、文化创意等新兴文化产业。整合文化产业资源，促进文化与旅游相融合，大力发展绢花、金镶玉、石人山珍等文化旅游关联产业。完善文化产业项目库，搞好文化产业招商引资，加强文化交流与合作。积极引导和扩大居民文化消费，加强文化市场监管。

(三)积极推动文化体制改革。加快文化管理体制改革，实现由办文化为主向管文化为主转变。继续深化图书馆、文化馆、广播电台、电视台等公益性文化事业单位内部人事、收入分配和社会保障制度改革。积极推动经营性文化事业单位转企改制，妥善解决资产管理、人员安置等问题。完善对重点文艺院团等 公益性文化事业单位的扶持方式。放开发展社会文化产业，鼓励引导非公有资本进入文艺表演、文化旅游、书报音像制品分销等文化产业领域，积极扶持民间职业演出团体和农村业余剧团，促进民营文化企业健康发展。

(四)进一步加强和谐文化建设。大力弘扬以爱国主义为核心的民族精神和以改革创新为核心的时代精神，深入开展社会主义荣辱观教育。以增强诚信意识为重点，加强社会公德、职业道德、家庭美德和社会信用体系建设。搞好廉政文化建设。加强和改进思想政治工作，注重人文关怀和心理疏导。做好未成年人、大学生法制和思想道德教育工作，普及青少年生理、心理、伦理教育。积极开展全民阅读活动。加强网络文化建设和管理。大力推进和谐社区、和谐校园、和谐家庭和文明行业、文明单位创建活动，加强农村文化、社区文化、广场文化等群众文化建设，广泛开展科教、文体、法律、卫生进社区和文化、科技、卫生“三下乡”活动，不断提高全社会文明程度。

五、着力加快以改善民生为重点的社会建设

在经济发展的基础上，着力保障和改善民生，努力使全市人民学有所教、劳有所得、病有所医、老有所养、住有所居。

(一)优先发展教育事业。全面实施城乡免费义务教育。农村义务教育在免除学杂费的基础上，全部免费提供教科书;城市义务教育从秋季起全部免除学杂费。筹措资金1.7亿元，提高农村义务教育阶段中小学公用经费标准和贫困家庭寄宿生生活费补助标准。继续推进城镇薄弱中小学改造和农村中小学危房改造，实施农村中小学图书教学仪器设备充实工程，进一步改善办学条件。开展城镇教师支援农村教育工作，从市属学校选派100名教师到农村支教。大力发展职业教育，整合职业教育资源，加强职业教育基础能力建设，把职业教育与农民工培训相结合，着力培养技能型人才。努力提高高等教育质量，促进高等教育办出特色、办出水平。以市卫生学校为依托，积极筹建平顶山医学高等专科学校。鼓励和规范民办教育发展。完善教育资助体系，着重保障经济困难家庭子女平等接受义务教育，继续实施高中阶段贫困生救助制度。用两年时间基本完成农村义务教育“普九”债务清理化解工作。认真解决农民工子女就学和城镇中小学大班额问题。

(二)大力发展医疗卫生事业。加强农村三级卫生服务网络建设。筹措资金4531.5万元，再改造4所县级医院，为29所前期投入不足的贫困乡(镇)卫生院配备基本医疗设备，完成全市村卫生室标准化建设，选派190名优秀医师到95个乡(镇)卫生院工作。加快城市社区卫生服务体系建设，健全社区卫生服务财政补 助机制。抓好重大传染病预防控制，继续做好地方病防治、慢性病防治和计划免疫工作。扶持中医药事业发展。筹措资金1181万元，开展创建白内障无障碍市“曙光行动”。强化卫生应急队伍培训，提高疾病预防控制和卫生应急能力。组织县级以上医院参加全省统一药品网上招标采购，实现同种药品全市同价。规范医疗收费价格，进一步扩大按病种付费试点。加强对合作医疗定点服务机构的监管，让群众得到适宜、价廉、安全、质优的医疗服务。

(三)积极做好就业和收入分配工作。认真贯彻就业促进法和劳动合同法，完善创业服务体系，优化创业环境，形成千家万户创业的局面，以创业带动就业。继续推进创业孵化园区建设，加大就业援助力度，实现下岗失业人员再就业2.5万人，其中就业困难人员再就业1万人，动态消除“零就业家庭”。做好高校毕业生、退伍转业军人、残疾人就业工作。实行职工带薪休假制度。加强劳动争议处理和劳动保障监察，严厉打击各种非法用工行为。解决好拖欠职工工资和农民工工资问题。深化收入分配制度改革，建立职工工资正常增长和支付保障机制，逐步提高最低工资标准。

(四)不断提高社会保障能力。做好城镇企业职工养老保险省级统筹衔接工作，继续做实个人账户。从今年起，再连续三年提高企业退休人员养老金水平，今年筹措资金9130万元，人均月增加103元。推进事业单位养老保险制度改革，探索建立农村养老保险制度。筹措资金3280万元，健全城镇居民基本医疗保险制度。筹措资金3.07亿元，将新型农村合作医疗的参合标准由每人每年50元提高到90元。积极将高风险行业从业人员纳入工伤保险覆盖范围。加强社保资金监管，确保基金安全。继续完善城乡医疗救助制度，将血友病、白血病等特殊疾病纳入医疗救助范围。筹措资金1.54亿元，将城市低保人均月补差标准由85元提高到110元，农村低保每人每月补差标准由30元提高到不低于40元，逐步将农村符合条件的贫困人口全部纳入低保范围。筹措资金3800万元，加强农村敬老院建设，提高农村五保供养标准，将集中供养率提高到40%。大力发展以扶老、助残、救孤、济困为重点的社会福利和慈善事业。继续办好爱心救助超市。调整住房供应结构，大力发展省地节能环保型住宅，增加中低价位、中小户型普通商品房供应。筹措资金4200万元，开工建设廉租房2万平方米，发放廉租房补贴1466万元。落实经济适用房建设用地，开工建设经济适用房15万平方米，竣工5万平方米。筹措资金14.75亿元，全面完成平煤集团棚户区改造任务。发放住房公积金贷款3亿元，帮助3000个家庭解决住房困难。落实好稳定物价的各项政策措施，加强价格监管，保持市场物价基本稳定。完善对低收入群众补助办法，切实保障困难群众基本生活水平不因涨价而下降。

(五)进一步强化安全生产监管。突出抓好煤矿、非煤矿山、危险化学品、民爆物品、烟花爆竹、特种设备等重点领域的安全生产，强化安全隐患排查专项治理，坚决遏制重特大事故发生。加强道路交通安全管理，强力整治道路运输超限超载行为。开展食品安全专项整治，使全市城市、农村食品抽检合格率分别提高 2个和4个百分点。加快建设农村药品监督网和供应网，全市所有乡(镇)建立食品药品安全协管站，药品配送进村率95%以上。积极探索并建立政府统一领导、部门依法监管、企业全面负责、社会监督支持的安全生产长效机制。加强监管队伍建设，落实监管日志制度，确保各级政府的监管责任和企业的主体责任落到实处。

(六)切实加强社会管理。进一步完善城市社区功能，继续开展农村社区建设试点。依法做好第六届村委会换届选举工作。加强信访工作，有效预防和化解各类社会矛盾。改革和加强城乡社区警务工作。加强法制宣传教育，积极推进普法依法治理工作。深入开展平安创建活动，健全社会治安综合防控体系，依法严厉打击暴力性犯罪、经济犯罪和盗窃、抢劫等多发性侵财犯罪。继续加强市长热线网络建设。坚持计划生育基本国策，统筹解决人口问题。稳定低生育水平，提高出生人口素质，综合治理出生人口性别比偏高问题。全面实施计划生育家庭奖励扶助制度和困难家庭救助制度。加强流动人口计划生育服务管理。坚持政府与工会联席会议制度，及时解决职工普遍关心的热点难点问题。做好法律服务和法律援助工作。重视发展老龄事业，切实保障妇女和未成年人权益，关心和支持残疾人事业。

深入开展国防教育，支持驻平解放军和武警部队建设，不断提高人民防空和民兵预备役的应急应战能力。认真落实军人优待抚恤政策，巩固扩大双拥成果。做好企业军转干部维稳解困工作。全面贯彻党的民族宗教政策，发挥宗教界人士和信教群众在促进经济社会发展中的积极作用。发挥工会、共青团、妇联等人民团体的桥梁纽带作用。继续做好外事、侨务、对台、史志、档案、盐业、地震、通信、国家安全等工作。

六、积极推进生态文明建设

加大生态建设投入，强化节能降耗和环境保护，着力建设资源节约型和环境友好型社会。

(一)加快林业生态建设。积极实施林业生态市建设规划，以工程项目造林、生态廊道建设和城郊绿化为重点，充分利用现有土地空间，优化林业生态建设布局，加快造林绿化步伐，力争所有县(市、区)2012年前建成林业生态县。实行行政首长负责制，严格目标考核奖惩。继续实施国家重点生态工程，突出抓好我市10大重点生态防护林工程和3大林业产业工程。拓宽融资渠道，增加财政投入，鼓励社会资金投入。筹措资金3.99亿元，完成大面积造林21.75万亩，新建和完善农田林网43万亩，完成生态廊道绿化1547公里，中心城区新增绿地2200亩。郏县建成省级林业生态县，再完成20个市级林业生态乡、300个林业生态村建设任务。

(二)加强能源资源节约。认真落实节能减排统计、监测、考核实施方案和办法，严格实行节能减排责任制和“一票否决”制。落实财税优惠政策和价格政策，实行以奖代补、强制政府采购等措施，促进节能减排。进一步淘汰落后产能，推动焦化行业深度治理工作。严格执行新建项目节能环保准入标准。加强 重点领域、重点行业和重点企业节能，着力抓好年综合能耗5000吨标准煤以上重点企业的节能工作。加快节能技术研发与推广，大力推进燃煤工业锅炉改造、热电联产、余热余压利用等重点节能工程建设，实施建筑节能改造示范工程。加强水资源节约和保护，认真落实建设项目水资源论证和节水设施“三同时”制度。继续开展“空心村”、砖瓦窑厂、工矿废弃地专项整治，完成已关闭砖瓦窑厂土地复垦工作，力争全年盘活存量建设用地1800亩以上。积极推广新型墙材，全面禁止使用实心黏土砖。

(三)大力发展循环经济。制定循环经济发展规划，全面推进煤炭、电力、食品、化工及农业、林业、畜牧业等领域的循环经济发展。积极推进示范企业、示范技术、示范园区等循环经济示范工程建设，着力建设煤盐化工、煤电建材等循环经济工业园。研究出台利用煤矸石、粉煤灰、电石渣等发展新型建材的规划，积极推广水泥行业低温余热发电、城市中水回用、矿井瓦斯抽采、发电余热制冷等先进技术，支持秸秆等生物质能源和材料的资源利用。着力培育一批再生资源循环利用企业，大力推进废旧资源再生利用。力争新型墙材产能达到34亿标块，工业固体废弃物综合利用率达到72%以上。

(四)强化环境综合整治。全面完成污染源普查工作。加强饮用水源地水质安全建设，探索并建立昭平台、白龟山水库及周边湿地环境保护的长效机制。加快燃煤电厂除尘脱硫设施建设，各类燃煤机组年底前全部完成脱硫设施建设并达标稳定运行。完成所有焦化企业消烟除尘、化产回收、污水处理等综合整治任务。切实做好省级污水处理示范区建设，确保污水处理平均负荷率达到75%以上。加快建立和完善城乡垃圾收集清运处理系统，积极推进城乡垃圾无害化、产业化处理。实施农村小康环保行动计划，控制和治理农村面源污染。加大矿山生态恢复治理力度。通过努力，城区大气环境质量优良天数达到300天以上，城市集中饮用水源地水质达标率100%，出境水基本达标。

七、进一步提高政府管理和服务水平

创新体制机制，加快法治诚信服务型政府建设，提高工作效率和服务水平，建设人民满意的政府。

(一)坚持依法行政。严格执行人大及其常委会的决议、决定，自觉接受人大及其常委 会的法律监督、工作监督和政协的民主监督，广泛听取各民主党派、工商联和无党派人士的意见和建议，认真办理人大代表建议和政协提案。依法规范行政许可、工程招投标、财政资金使用、政府采购、国有资产转让等公共资源管理权力的运行，严格按照法定权限和程序行使权力、履行职责。坚持用制度管权、管事、管人，加强对领导干部、人财物管理使用和关键岗位的监督，建立健全质询、问责、经济责任审计、引咎辞职等制度。深入开展规范行政处罚自由裁量权活动，落实执法责任制和执法过错追究制。畅通行政复议渠道，依法化解行政争议。

(二)提高行政效率。深化行政管理体制改革，健全部门之间协调配合机制，着力解决职能交叉、权责脱节、政出多门的问题。完善重大事项集体决策、专家咨询、社会公示与听证等制度，推进决策的科学化、民主化。深化行政审批制度改革，进一步减少和规范行政审批事项。继续整合行政审批职能，各单位行政审批事项向行政审批服务大厅集中，单位内部向一个科室集中，实行一个“窗口”对外。压缩审批时限，使行政审批事项的办理效率总体走在全省前列。

(三)强化服务职能。加快转变政府职能，在加强和改善经济调节、市场监管的同时，更加注重履行社会管理和公共服务职能，努力建设服务型政府。完善公共服务体系，优化公共资源配置，将公共资源更多地向公共服务领域倾斜，维护社会公正和社会秩序，促进基本公共服务均等化。认真落实政府信息公开条例，积极推行学校、医院、供水、供电等公用事业单位办事公开制度，保障人民群众的知情权、参与权和监督权。加快电子政务建设，建立健全政府门户网站。加强统计监督，提高服务水平。做好气象灾害特别是极端天气事件的监测、预警。强化应急管理体系建设，有效应对自然灾害、事故灾难、公共卫生和社会安全事件，提高危机管理和抗风险能力。

(四)加强政风建设。坚持为民、务实、清廉，切实增强忧患意识、节俭意识、公仆意识，进一步转变思想作风和工作作风。健全政府绩效考核体系，把各方面工作引导到科学发展、和谐发展轨道上来。大兴调查研究之风，真诚倾听群众呼声，真情关心群众疾苦，涉及群众利益和公共政策的重大事项要广泛听 取意见。大力弘扬求真务实、真抓实干的作风，坚持讲实话、办实事、求实效，不搞花架子，不做表面文章，认认真真、扎扎实实地开展工作。反对以会议贯彻会议、以文件落实文件，提倡开短会、讲短话，力戒形式主义和官僚主义。牢记“两个务必”，坚持勤俭节约，严格财政支出管理和公费出国出境管理，规范公务接待行为，坚决反对铺张浪费和奢靡之风。加强反腐倡廉建设，搞好廉政和效能监察，建设廉洁政府。继续开展优化经济发展环境集中整治活动，严肃查处“吃、拿、卡、要”和“四乱”行为，为企业依法生产、依法经营创造宽松环境。认真解决安全生产、环境保护、教育收费、土地征收征用与房屋拆迁等方面群众反映强烈的问题，坚决纠正损害群众利益的不正之风。深入治理商业贿赂，依法严惩腐败分子。加强对公务员的培训、管理和监督，努力建设一支高素质的公务员队伍。

各位代表！平顶山已站在新的发展起点上，人民的期望不允许我们有丝毫懈怠，时代的要求召唤着我们奋发进取。让我们在省委、省政府和市委的正确领导下，同心同德，开拓进取，扎实工作，埋头苦干，为谱写全市人民美好生活新篇章而努力奋斗！
